# Investigation of Eye-Tracking Scan Path as a Biomarker for Autism Screening Using Machine Learning Algorithms

**DOI:** 10.3390/diagnostics12020518

**Published:** 2022-02-17

**Authors:** Mujeeb Rahman Kanhirakadavath, Monica Subashini Mohan Chandran

**Affiliations:** 1 School of Electronics Engineering, Vellore Institute of Technology, Vellore 632014, India; m.rahman@ajman.ac.ae; 2 Department of Biomedical Engineering, Ajman University, Ajman P.O. Box 346, United Arab Emirates; 3 School of Electrical Engineering, Vellore Institute of Technology, Vellore 632014, India

**Keywords:** ASD screening, autism spectrum disorder, machine learning, convolutional neural network (CNN), eye-tracking scan path images

## Abstract

Autism spectrum disorder is a group of disorders marked by difficulties with social skills, repetitive activities, speech, and nonverbal communication. Deficits in paying attention to, and processing, social stimuli are common for children with autism spectrum disorders. It is uncertain whether eye-tracking technologies can assist in establishing an early biomarker of autism based on the children’s atypical visual preference patterns. In this study, we used machine learning methods to test the applicability of eye-tracking data in children to aid in the early screening of autism. We looked into the effectiveness of various machine learning techniques to discover the best model for predicting autism using visualized eye-tracking scan path images. We adopted three traditional machine learning models and a deep neural network classifier to run experimental trials. This study employed a publicly available dataset of 547 graphical eye-tracking scan paths from 328 typically developing and 219 autistic children. We used image augmentation to populate the dataset to prevent the model from overfitting. The deep neural network model outperformed typical machine learning approaches on the populated dataset, with 97% AUC, 93.28% sensitivity, 91.38% specificity, 94.46% NPV, and 90.06% PPV (fivefold cross-validated). The findings strongly suggest that eye-tracking data help clinicians for a quick and reliable autism screening.

## 1. Introduction

Autism spectrum disorder (ASD) is a neurodevelopmental disorder characterized by deficits in verbal and nonverbal communication, reciprocal social interaction, accompanied by certain repetitive and stereotyped behaviors [1]. The severity of symptoms and the effects of ASD varies from case to case. According to the Centers for Disease Control and Prevention (CDC), 1 in 54 children has been diagnosed with ASD; it affects people of all races, ethnicities, and socioeconomic backgrounds. Moreover, ASD is diagnosed four times more frequently in boys than in girls; compared to boys, many girls with ASD show fewer visible signs [2]. Autism is a chronic illness that lasts a lifetime [3]. Therefore, early identification of ASD is crucial, and individuals with ASD diagnosed early in childhood can significantly impact from appropriate interventions for a long-term positive outcome [4].

We currently lack a diagnostic test to confirm ASD, such as a blood test or a brain scan. A behavioral assessment would be required to diagnose ASD in a child. The American Academy of Pediatrics (AAP) recommends that all children be screened for developmental delays and disabilities at 9, 18, and 30 months during routine doctor visits. However, at 18 and 24 months, every child should be screened for ASD using autistic-specific screening tools based on the Diagnostic and Statistical Manual for Disorders—DSM_V [5].

ASD diagnosis is still a challenging task requiring several cognitive tests and hours of clinical examinations and follow-up. Furthermore, the heterogeneities in the symptoms of ASD make the diagnosis even more difficult [6]. Autistic children exhibit symptoms from a young age but are frequently not diagnosed until school age. A few studies have identified a lack of resources in rural areas and low-income families, the efficacy of ongoing pediatric care, a shortage of specialty referrals, and the parents’ awareness of ASD as possible causes of diagnosis delays [7].

It is a fact that individuals with autism pay less attention to faces and other social stimuli such as the human voice and hand gestures when compared to nonsocial stimuli [8,9]. This unique pattern of eye movements appears in autistic children well before behavioral symptoms of ASD start showing. Using the eye gaze data, we can figure out when the child is looking and what they are looking at, whether it is people or objects. Furthermore, the eye gaze data are instrumental in determining how difficult it is for an autistic to interact with others, allowing interventions to be tailored to their specific needs.

Eye-tracking (ET) is a non-invasive technique for recording a person’s gaze positions in real-time. An eye-tracking device is mainly composed of a high-resolution digital camera and an intelligent algorithm that accurately detects eye gaze coordinates while watching videos or images. According to relevant studies, ET data may be used as a biomarker for identifying ASD in children at an early stage [10].

Machine learning (ML) has recently gained traction in various fields, including medical diagnosis [11,12,13,14]. ML is a subset of Artificial Intelligence (AI) that employs intelligent algorithms to learn patterns from large datasets (called training an ML model) without explicit programming. To make an informed decision on new data, the model uses the learned knowledge in the same way the human brain works. There are two common learning approaches in machine learning: Supervised and unsupervised, depending on the type of dataset used to train the model. Supervised models require labeled datasets to categorize the data into binary or multi classes—also called classification or regression. In comparison, the unsupervised models deal with unlabeled data and allow the system to detect patterns within the dataset on its own (called clustering).

Deep Learning (DL) is a subset of machine learning that makes use of specialized algorithms called artificial neural networks (ANN) to simulate the human brain [15]. The Deep Neural Network (DNN) is an extensive network of artificial neurons capable of handling complex problems [16]. A convolutional neural network (CNN) is an ANN that has the capability to learn features from signals and images autonomously and adaptively [17]. CNN finds their applications in image processing, such as object detection, object recognition, anomaly detection, and segmentation [18]. CNN’s main advantage over its predecessors is that it extracts relevant features from the input image without the need for human intervention.

In this study, we utilize machine learning algorithms to investigate the efficacy of eye-tracking data in diagnosing autism in children. Unlike the existing manually administered autism screening procedures, which are highly subjective and thus prone to misdiagnosis, our research uses eye-tracking data to detect autism quickly and reliably. Instead of the 1D eye-tracking time series data utilized in related investigations, we use 2D eye-tracking scan path (ETSP) images. We employ principal component analysis (PCA) and CNN as feature extractors and the boosted decision tree (BDT), deep support vector machine (DSVM), decision jungle (DJ), and deep neural network (DNN) as classifiers. In contrast to the previously reported studies, we evaluate the model performance using the area under the ROC curve (AUC), specificity, sensitivity, positive predictive value (PPV), and negative prediction value (NPV).

## 2. Literature Review

We have reviewed relevant literature on the above topics and presented the most recent ones here. Almourad M. [19] collected eye-tracking data from 65 participants, 34 of whom had been diagnosed with ASD and 31 of whom were typically developing (TD). The average age of the participants was eight years old. The data were collected using a Tobi X2 eye-tracking device. While the experimenter recorded their eye fixation, the kids sat in front of the eye tracker and watched the objects (such as tomato, football, banana, and a human child). In this study, children with autism had less fixation on their eyes and a greater interest in gazing at the mouth than TDs.

In a similar study reported by Duan et al. [20], the researchers recorded the eye movements of 14 autistic children and 14 TDs to create a dataset. There was a total of ten sessions in this study, each with 30 images (300 natural scenes). Each image is displayed on the screen for 3 s before being replaced by the following image after 1 s. Data on gaze were collected using the Tobii T120 eye tracker. According to the study, people with ASD have more fixation on objects, whereas people with TD have more fixation on faces.

Tao et al. [21] used CNN to classify ASD and TD based on the scan path of the fixation point in their study. They used 300 images from the ‘Saliency4ASD’ grand challenge dataset, including 14 autistic and 14 neuro-typical. The authors proposed a framework called SP-ASDNet for autism screening based on the observer’s gaze scan paths. To extract the features from the saliency map, the authors adopted CNN-LSTM architecture. The proposed model achieves 74.22% accuracy on the validation dataset. The authors intend to include an attentive mechanism in future studies and integrate it into the model to improve performance. According to the authors, it is necessary to conduct more research into the scan path differences between ASD and TD.

Carette R. et al. [22] published a paper that presents experimental results using an ML approach to detect autism spectrum disorder automatically. The children, aged 8 to 10, were divided into autistic (class 1) and TD (class 2). Each child showed a pre-recorded video of a joint attention offer, and an eye-tracking system records all eye movements. The authors used an LSTM-RNN based ML classifier to test the recordings. The model correctly identified five of the six patients’ diagnoses from fitness values of 0.008 to 0.006 and scored an average accuracy of over 90% and a maximum of 98%. The investigation reveals a sensitivity of 75% and a specificity of 100% in the best-case scenario. The authors recommend that using other ML algorithms may help improve the prediction score. They also mentioned the possibility of model overfitting due to the small dataset.

In the same year, Carette R. et al. [23] published another interesting research paper that discussed the use of eye-tracking technology in the context of ASD. The authors presented a method for converting eye-tracking records into eye-tracking scan path images as a visual representation using color gradients, which is well-suited for Machine Learning. The authors also provided a publicly available first-of-its-kind image dataset based on a series of eye-tracking experiments with ASD diagnosed (*n* = 30) and TD (*n* = 29) participants. Using an SMI RED series mobile eye tracker, the authors recorded ET data while the participants watched a series of videos to stimulate eye movement across the screen. In their second publication, the authors mentioned above provided the findings of several classical and neural network (NN) based ML classifiers to identify ASD using the pre-recorded eye-tracking scan path dataset [24]. The classical algorithms include Naïve Bayes, Logistic Regression, SVM, and Random Forest. According to the obtained result, the logistic regression model was the best performing conventional model, with an AUC of 70%, whereas the NN model achieved an AUC of 90%. Furthermore, the authors developed a four-level severity-based classifier model for ASD, with classes such as TD, low, medium, and severe based on Childhood Autism Rating Scale (CARS) score, with a maximum classification accuracy of 93% for TDs and 6% for Severe ASD. According to the authors, the size of the dataset and the limited duration of input videos (used as visual stimulus) in their trials were two significant limitations of this study.

Recently, Oliveira et al. [25], collected ET data from 106 subjects using Tobii Pro TX300 equipment, with 30 participants belonging to the TD group (10 females and 20 males) and 76 belonging to the ASD group (27 females and 49 males), ranging in age from 3 to 18 years. ET data were collected using a 54-second video with nine randomly selected visual stimuli parts (each with a child’s interaction and a geometric movement). The authors employed a genetic algorithm to identify the ASD-relevant traits from the ET data and chose 15 features that the classifiers (SVM and ANN) require. The paper documented that the SVM model scored an AUC of 77.5%, while the AUC of the ANN model was 82.2%. The authors faced a significant challenge in obtaining annotated datasets from experts, which is problematic. The authors were willing to test the existing dataset with other machine learning techniques in future work.

In summary, the above literature identified that autistic children exhibit unique eye gaze patterns compared to age-matched TDs, making ET data a viable biomarker for ASD screening. Some researchers utilized state-of-the-art technology to obtain ET data accurately directly from the children; however, this necessitates costly equipment, experimental setup, and an adequate number of participants for trials. Pre-recorded datasets freely accessible to the public strongly influence the relevant research; however, many authors reported the shortage of a comprehensive ET dataset well suited for ML-based research work. Despite the numerous attempts, the clinical utility of ML algorithms is still a distant dream. To our knowledge, the most up-to-date research model had a maximum accuracy of 90%; However, the authors did not mention about sensitivity, specificity, PPV, NPV, and AUC of the model. Therefore, this research study focuses on finding the optimal algorithms for identifying ASD in children using the visualized eye-tracking scan path images, and also on making this paper as straightforward as possible to convey the essential concepts of ML to an autistic specialist who is unfamiliar with the field of ML.

## 3. Materials and Methods

### 3.1. Data

**Figure 1:**
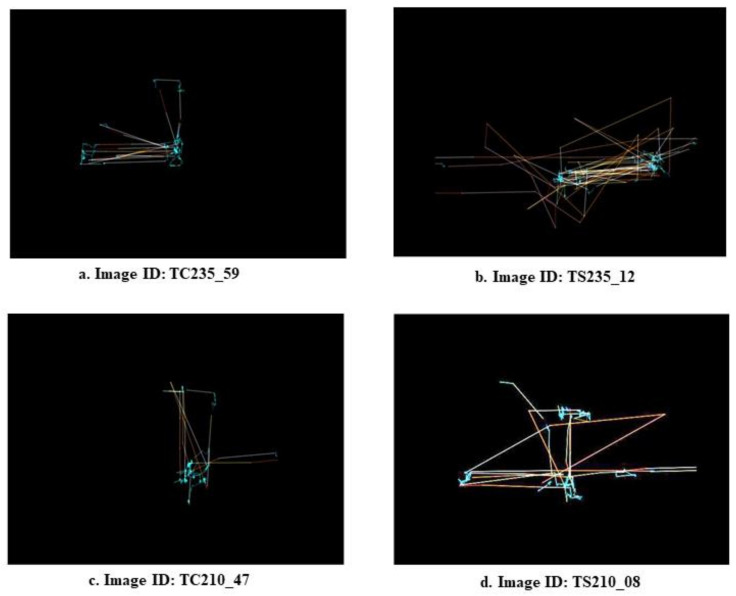
ETSP images from the dataset as examples (images on the left side for a TD participant and images on the right side for an ASD diagnosed participant—Image ID’s as given in the dataset).

We discovered an exciting dataset contributed by Mahmoud E. [26], as mentioned in the Section 2, while searching for a reliable dataset for the early screening of ASD. This dataset contains 547 visualized Eye-Tracking Scan Path (ETSP) images created from ET data collected from 59 participants in an experiment using a series of autism-specific visual stimuli using an SMI RED eye tracker (https://imotions.com/hardware/smi-red-m/, accessed on 12 January 2022). The authors created visualized images by extracting three dynamic components from the ET data, namely, velocity, acceleration, and jerk, and mapping them into three equal-sized planes (RGB). The values of the three components modulated the gradient of colors in each channel. Table 1 summarizes the dataset’s most important details, and Figure 1 shows two sample images from both classes (TD and ASDs).

### 3.2. Overview of the Proposed ML Model

**Figure 2:**
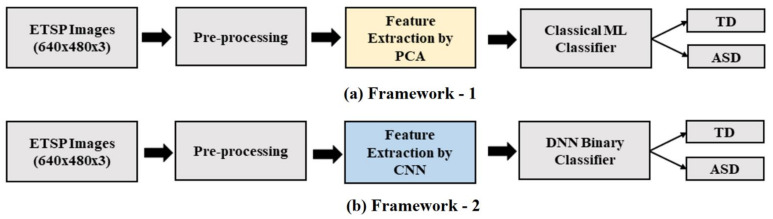
The framework of the proposed model.

As depicted in Figure 2, the proposed ML model, composed of framework-1 and framework-2. Many operations in the frameworks are the same, except the feature extraction, which is considered a critical step.

Below are descriptions of the various stages (a to f) and relevant processes:

(a) Pre-Processing

The first step in the pipeline is image pre-processing, and it consists of two distinct operations: RGB to grayscale conversion and image resizing. The input image has three channels and is 640 × 480 pixels (Red, Green, and Blue). We use Equation (1) to convert the RGB image to grayscale, where R, G, and B denote pixel data from the Red, Green, and Blue channels, respectively. We then resize the resulting grayscale images of size 640 × 480 to 100 × 100. This step reduces the computational load on the computer, allowing for faster computation.

Grayscale Image = R/3.0 + G/3.0 + B/3.0(1)

(b) Feature Extraction by PCA

PCA (Principal Component Analysis) is a popular feature extraction method in machine learning algorithms. PCA identifies the relationship between the input raw data attributes and generates a feature map with relatively lesser dimensionality to present the same information associated with the given input data [27]. Thus, PCA reduces the dimensions of the data without losing information while generating feature maps for the raw data. As a result, using PCA in ML algorithms significantly impacts computing speed [28].

(c) Classical ML Classifier

This serves as the output of framework-1. The goal of the ML classifier is to accurately classify images into TD or ASD using the features extracted in the PCA stage. Classic machine learning algorithms such as Linear Regression (LR), Support Vector Machine (SVM), Decision Tree (DT), and random forest excel at solving a wide range of problems. These models are the most popular in machine learning because they are simple, ideal for small datasets, compatible with low-power computers, and computationally less expensive. To evaluate and compare the results of framework-1, we adopted Boosted Decision Tree (BDT), Deep Support Vector Machine (DSVM), and Decision Jungle (DJ). The following section provides a quick overview of the three classical algorithms.

BDT Algorithm

The Decision Tree (DT) algorithm uses a tree-like decision model to classify the data by applying specific criteria. It incrementally breaks down a dataset into smaller and smaller subsets while also developing an associated decision tree. The data splitting process continues until a particular condition of stop is reached. The DT became well-known in a variety of fields, including medical diagnostics. This approach would provide great accuracy in classification or regression issues with only a few criteria. Boosting is a technique for improving a weak classifier’s performance by narrowing the gap between the actual (true) and predicted values [29]. 

DSVM Algorithm

Support Vector Machines (SVM) is a supervised ML algorithm, most often used for classification problems; however, it can also be used for regression. The SVM algorithm fits partition boundaries between data points to separate various classes in a given dataset. To divide the distinct types in a dataset, one could construct many partitioning lines. On the other hand, SVM fits the best hyperplane between the two classes, i.e., the most significant margin between the hyperplane and the nearest data points on each side [30]. SVMs can readily classify data that is linearly separable; however, when dealing with data that is linearly inseparable, SVMs use kernel tricks to transform the 2D data into multi-dimensional spaces to find the best borders between classes. DSVM is an extension of SVM, which has a layered architecture consisting of SVMs at each layer. The SVMs at the low levels of the DSVM have support vector machines to capture relevant features from the input data and pass on the extracted data to the next level recursively. The SVMs on the top of the model use the highest-level extracted features as inputs to perform the actual prediction. A two-layer DSVM consistently outperforms a regular SVM in performance [31].

DJ Algorithm

Random Forest (RF) and DT are flourishing for solving many practical applications, particularly computer vision. However, these algorithms experience some fundamental issues: the number of nodes will grow exponentially in DT algorithms when data are fed to the classifier, creating a requirement for additional memory, and thus impacting the model’s performance [32]. Decision Jungle employs decision-directed discriminative models using DAGs (Decision-directed Acyclic Graphs) to achieve the potential to solve complex problems without storage issues [33]. The DAGs allow multiple ways from root to leaf to simplify the classification task. The DT allows only one path to every node in comparison to DJ. Results indicate that compared to RF or DT, the DJ has less memory requirement, and thereby it affords considerable improvement in the model’s generalization.

(d) Feature Extraction by CNN

**Figure 3:**
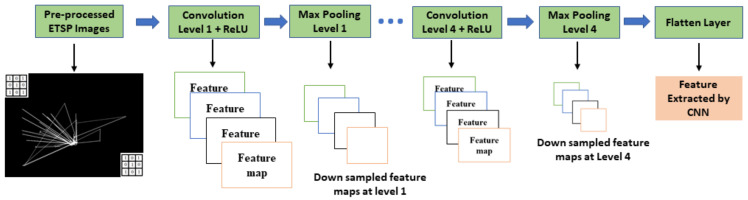
Structure of a four-layer CNN feature extractor.

Framework-2, as previously mentioned, uses CNN to extract essential features from pre-processed ETSP images. The structure of the CNN feature extractor that we used in framework-2 is in Figure 3. It primarily consists of four alternate convolutional and max-pooling layers, marked as Level 1 to Level 4. Convolution layers consist of many kernels, each performing a mathematical operation known as convolution, which extracts features from digital images [34,35].

The default dimension of the kernel is 3 × 3; however, 5 × 5 or 7 × 7 kernels are also in use. Each kernel location carries some coefficients called weights; weights are learnable parameters of a CNN, and its value keeps updating as the model learns from training data. Equation (2) defines convolution, where *f*(*x*,*y*) is a digital image, *w*(*x*,*y*) is a kernel, *m*, and *n* define the kernel size [34].

(2) $$\left(x,y\right)\;=f\left(x,y\right)\;*\;w\left(x,y\;\right)\;=\overset{m}{\underset{t=-m}{\sum }}\overset{n}{\underset{h=-n}{\sum }}f\left(t,h\right)w\left(x-t,y-h\right)\;$$

The image and kernel are convoluted to yield an output matrix *g*(*x*,*y*) with the exact dimensions as the input image. An activation function converts the matrix into a feature map at the output. The activation function in our model is a rectified linear function (ReLU). This function returns *M*(*x*,*y*) the exact value of *g*(*x*,*y*) for all positive numbers in the output matrix and 0 otherwise as indicated in (3) [35]

(3) $$M\left(x,y\right)\;=\;max\;\langle 0,g\left(x,y\right)\rangle$$

The max-pooling layers are used in conjunction with convolutional layers to reduce the dimensions of the feature maps. This step is sometimes called down-sampling [35]. The max-pooling layer calculates the feature map’s maximum value for each patch. We used max-pooling layers 2 × 2 in size, resulting in a 50% reduction in the feature map’s dimensions. The feature maps obtained at Level 1 are fed to the next level (Level 2) and follow the operation sequence until the final feature maps are available at Level 4.

All CNN-based classification models have a flatten layer available at the output terminal to reshape the output matrix and make it suitable for the next stage [34,35]. The spatial information from the features at level 4 is removed here and replaced with a channel dimension. As a result, unique channels are formed for each of the retrieved feature maps [36].

(e) DNN Classifier

**Figure 4:**
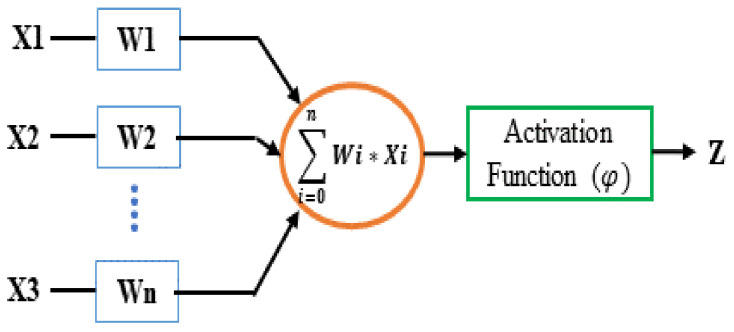
Basic Structure of a Neuron.

The last part of framework-2 is a binary DNN classifier to perform the classification by exploring the features extracted by the CNN block. DNNs are a feedforward network in which the data move from the input layer to the output via several hidden layers (Bengio et al. 2009). DNN uses many artificial neurons to simulate how the human brain solves problems. An artificial neuron has several inputs (dendrites), a processing unit, and an output (axon), as shown in Figure 4. As depicted in Figure 4, input information (Xi) and some weights (Wi) attached to each input will produce an output (Z) of 0 or 1. A neuron triggers if the cumulative sum of the input-weight product exceeds a threshold and produces ‘1’ at its output. The activation function performs the thresholding. The output of one neuron propagates to the input of another layer through a node (or synapse) [37,38]

**Figure 5:**
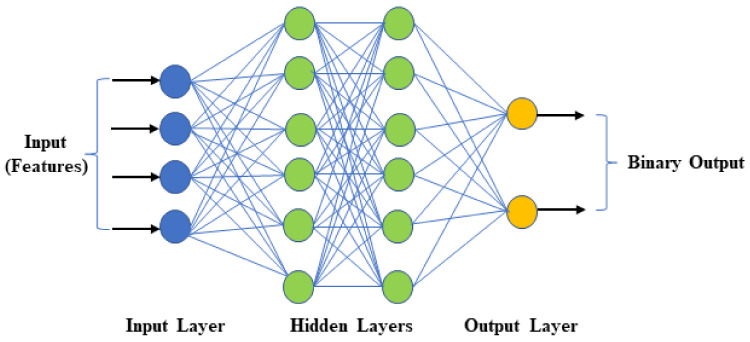
Structure of DNN binary classifier.

DNNs have multi-layered hierarchical structures with 1000 s of neurons. The number of hidden layers in the network varies depending on the problem. Some problems only require a single hidden layer, while others require multiple layers [39,40]. The DNN binary classifier shown in Figure 5 has an input layer that brings in flattened feature maps to the network, followed by two hidden layers to derive optimal mathematical functions that fit feature maps to the target output. Finally, an output layer makes final predictions (binary) based on the information received from the preceding hidden layers.

(f) Building the ML Models

The three ML models used in framework-1 (BCT, DJ, and DSVM) are easy-to-use built-in models available in Microsoft AZURE Machine Learning Studio (https://studio.azureml.net, accessed on 5 December 2021) and do not require any configuration settings. However, the CNN and DNN models used in framework-2 are handcrafted, and we tried various configurations to choose the optimal model. 

The dataset used to train a DNN-based ML model significantly impacts its generalization. A small dataset makes the algorithm learn quickly during training; however, the model cannot accurately predict unseen test data because of high variance in the test data. This situation is also called the overfitting of the model. DNN models become more skilled and capable of making accurate predictions with the help of a large dataset. Image augmentation is a commonly used method to populate a small dataset artificially. This technique creates duplicates of every image in the dataset by applying one or more of the following operations: rotation, translation, and cropping. By incorporating augmentation into the training dataset, the model obtains variants of images in the original dataset to learn from and make predictions with higher accuracy [41,42,43]. 

Our research used two types of datasets: the original dataset, which contains 547 ETSP images, and a large synthetic dataset, which was artificially created by applying augmentations to the images in the original dataset. The details of the dataset are provided in Table 2. 

We use the metrics Sensitivity, Specificity, Positive Prediction Value (PPV), Negative Prediction Value (NPV), and Area Under the Receiver Operating Characteristics (AUC) as given in Equations (4)–(7) to evaluate the models’ performance. Sensitivity is a metric that measures how frequently a test correctly generates a positive result for people who have the disease. Specificity is the ability of a test to correctly generate a negative result for people who do not have the condition. The PPV indicates the likelihood of having the disease after a positive test result; it is also called precision. The NPV shows the possibility of not having the condition after a negative test result. The Receiver Operating Characteristics (ROC) curve plots the True Positive Rate (TPR) against the False Positive Rate (FPR) at different threshold values. The AUC is a better estimate of accuracy because it summarizes the curve that measures a classifier’s ability to distinguish between classes [44].

(4) $$\mathrm{Sensitivity}\;=\frac{True\;Positives\;}{Total\;Positives\;}$$

(5) $$\mathrm{Specificity}\;=\frac{True\;Negatives\;}{Total\;Negatives\;}$$

(6) $$\mathrm{PPV}\;=\frac{True\;Positives\;}{Total\;Positives+False\;Postives\;}$$

(7) $$\mathrm{NPV}\;=\frac{True\;Negatives\;}{Total\;Negatives+False\;Negatives\;}$$

Cross-validating the model scores is critical to avoid overfitting in ML models when dealing with a small dataset. K-fold cross-validation (KCV) is a widely used method that divides the dataset into k folds to test the model ‘k’ times using ‘k’ independent training and test sets. The average of the ‘k’ test scores is the cross-validation score [45].

As previously stated, we used Microsoft AZURE Machine Learning Classic to create framework-1 models. On Google Collaboratory, we built a DNN-based classifier [46]. The goal of the Microsoft AZURE and Google Collaboratory is to promote machine learning research. The Collaboratory is a cloud-based Jupyter notebook environment that is free to use. Microsoft’s Machine Learning Studio (classic) is a GUI-based machine learning development platform for building ML models quickly. We used a Dell XPS 15 9500 laptop with a 2.60 GHz Intel(R) Core (TM) i7-10750H CPU, 6 Cores, and 12 Logical Processors as a local system. We used the following hyperparameters as default for both models: model loss—binary cross-entropy, number of training epoch-50: the batch size—32, and the learning rate—0.001 with an ‘Adam’ optimizer.

## 3.3. Experimentations and Results

We began our research with the original dataset, which, as previously stated, contained 547 ETSP images, each of size 640 × 480 × 3. To attain our objective, we planned and carried out a series of systematic and logical steps of research experiments that are described below in steps:

Step 1: Conversion of ETSP images to grayscale images and resizing

**Figure 6:**
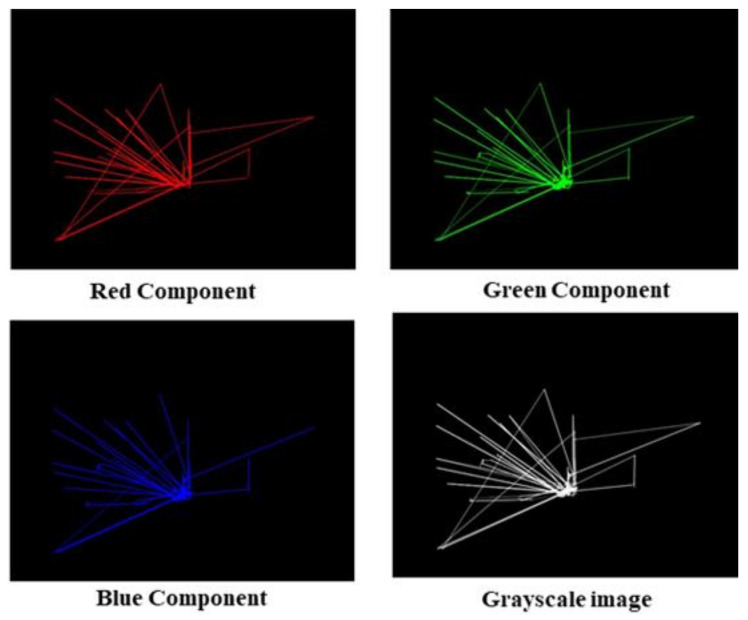
Three components (Red, Green, and Blue) of the ETSP and Grayscale Image.

Using the given Equation (1) in the Section 3, we converted these original images (RGB) to grayscale images of size 640 × 480. We treated the three color components equally by multiplying each by a factor of 1/3. Figure 6 shows Red, Green, and Blue components of the original image and the converted resulting grayscale image. To reduce the workload on the computer, we then resize the image to 100 × 100. We flattened the image array of size 100 × 100 into a dimension of 10,000 × 1 and appended class labels (0 for TD and 1 for ASD) to it. We repeated the above steps for all dataset images and stacked them into a 547 × 10,001 array before feeding them to the next stage. Note that the extra column in the stacked array stores the class labels as either 0 or 1.

Step 2: Application of PCA to Extract Features

**Figure 7:**
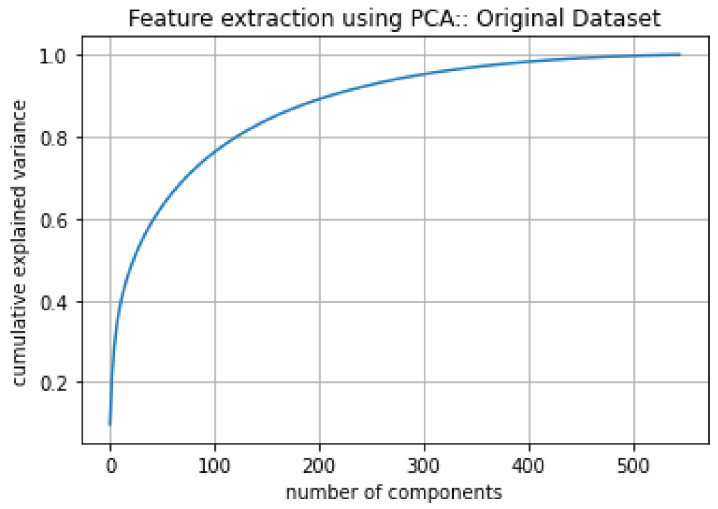
PCA analysis on original dataset.

The next step was to apply PCA on the stacked data to extract features. As input, PCA receives image data with a size of 547 × 10,001. The PCA identified 500 principal components for the given data, as shown in Figure 7, which serves as features for training the ML models.

Step 3: Creation of ML Model

We carried out the following procedures to create the ML models. We started by splitting the image data (547 × 10,000) into two parts: 70%:30%, with 382 images serving as the training set and 165 images acting as the model’s test set. We experimented with various classical ML algorithms to find the best algorithm for the given dataset. Table 3 shows the cross-validated (fivefold) scores of the top three best-performing models: DSVM, DJ, and BDT. 

Results in Table 3 show that the BDT algorithm performed better than the others, with a percent AUC 67.60%, a sensitivity of 64.4%, specificity 57.40%, NPV, and %PPV 59.60% (cross-validated with k = 5). Since the outcome did not appear promising, we decided to proceed with the DNN model.

Step 4: Design of DNN Models

At first, we built a DNN model with two convolution layers of 16 and 32, kernel size 3 × 3, pooling layer of size 2 × 2, flatten layer followed by one dense layer of size 128 and a sigmoid output layer. We continued modifying configurations of the above model, such as various combinations of convolutional, pooling, and dense layers until we found an optimal model. The optimal model has the following configurations as given in Table 4. The CNN module generated 4608 features from each pre-processed ETSP image (grayscale images of size 100 × 100) as input to help the classifier accurately predict ASD. Four convolution layers (16, 32, 64, and 128, with kernels of 3 × 3) with alternate max-pooling layers of size 2 × 2 and a flatten layer, the binary classifier with two dense layers (256 and 128) and one output layer with a binary sigmoid activation function. 

**Figure 8:**
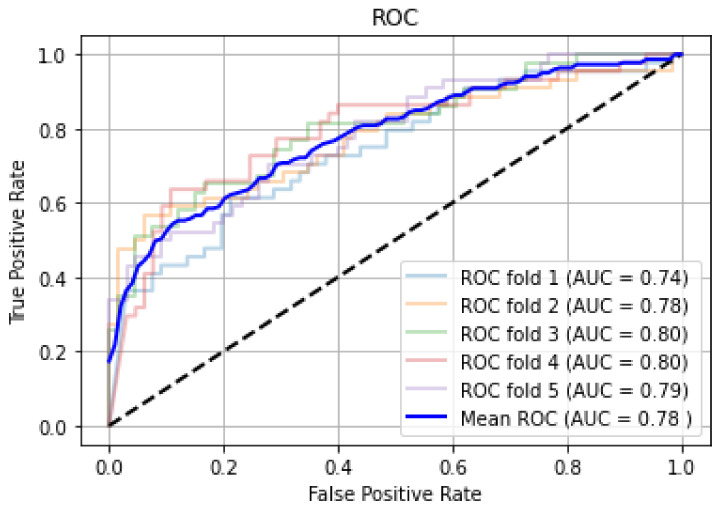
Cross-validated ROC on the original dataset.

We began the model’s training (70:30 data split) with default hyper-parameter settings (batch size: 32, learning rate: 0.01, number training epoch: 25), then fine-tuned the hyper-parameters until the model produced the highest score. The model achieved a mean AUC of 78% (cross-validated with k = 5) with sensitivity 78.57%, specificity 75.47%, PPV 87.12%, and NPV 62.5%. The corresponding hyperparameters were batch size: 16, Adam optimizer with a learning rate of 0.001, and the number of epochs: 50. Figure 8 shows the ROC curves CNN model for fivefold cross-validation; the CNN model achieved a relatively higher score on the original dataset.

Step 5: Creation of artificially populated more extensive dataset

**Figure 9:**
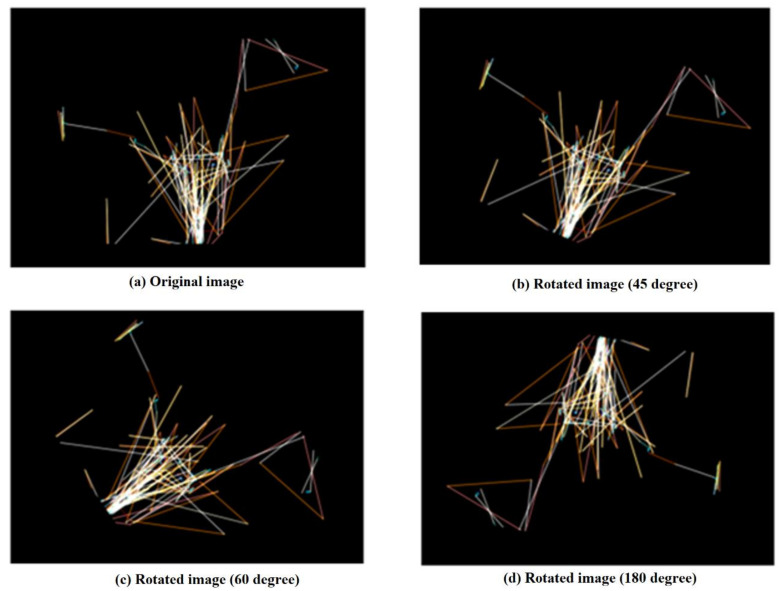
Three variants (**b**–**d**) of a sample image (**a**) in the original dataset generated by augmentation.

To attain higher performance, DNNs need a large amount of training data. Image augmentation is the most common but effective way to populate a small dataset. As explained in the Section 3, Image augmentation techniques apply one or more of the following operations such as shifts, zooms, random rotations, and flips on each of the images in the original dataset. Although the augmented images are a copy of the images in the original dataset, the orientation applied to them ensures that they never seem the same. We rotated each image in the original dataset by 45, 60, 90, and 180 degrees, resulting in a synthetic dataset of 2566 RGB images consisting of 1047 ASD and 1519 TDs. Figure 9 shows three augmented images generated by rotating a sample image in the original dataset at 45, 60, and 180 degrees. In the same order as the previous case, we continued the experiments on the synthetic dataset using the classic ML models (DJ, DSVM, and BDT) and the DNN model.

Step 6: Experimentations on the augmented dataset

**Figure 10:**
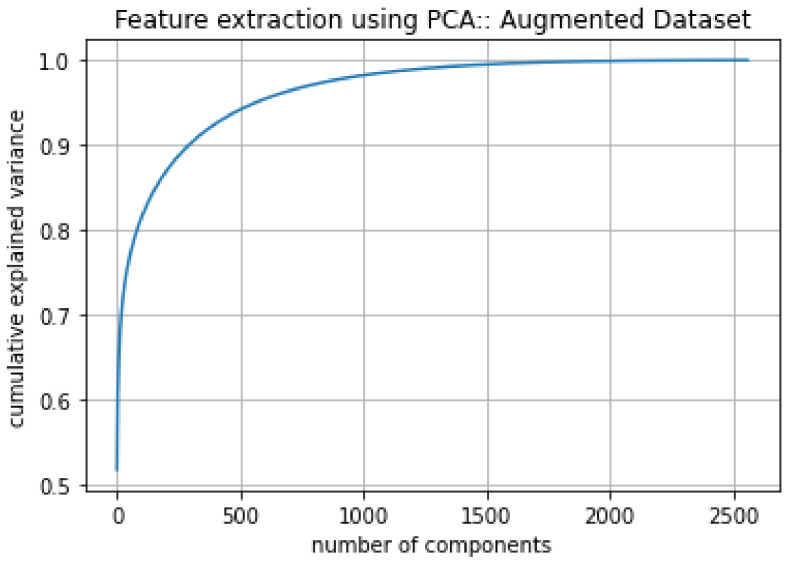
PCA analysis on the augmented dataset.

When we applied PCA on the augmented image data stack of size 2566 × 10,000, as demonstrated in Figure 10, the number of features reduced to 1750, resulting in an image array of 2566 × 1750. Table 5 shows the results of the three classic models on the augmented dataset. With this dataset, all models exhibited significant improvements in overall performance. Amon the classical models, BDT achieved the highest AUC of 74.60%, with a sensitivity of 49.80% and specificity of 86.5%. This model also reported a PPV of 70.56% and an NPV of 72.66% (all scores cross-validated with k = 5).

**Figure 11:**
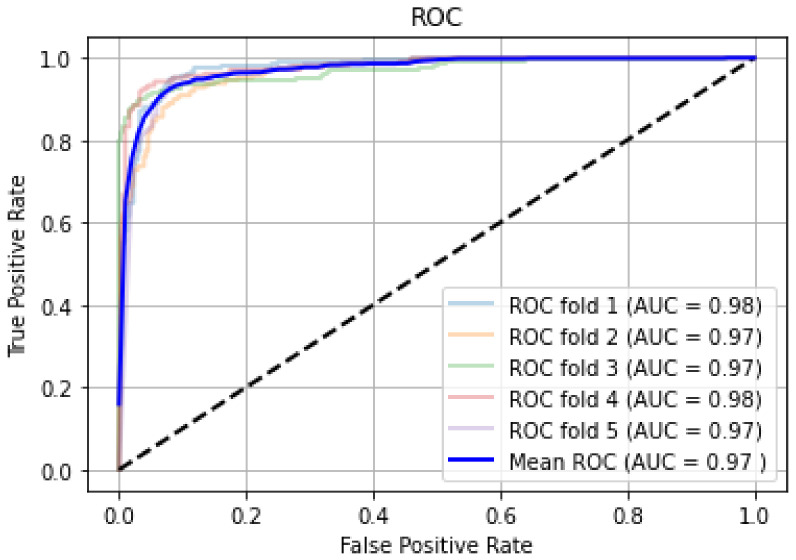
Cross-validated ROC on the augmented dataset using the CNN model.

When we trained the DNN model using the augmented dataset and performed the tests, this model achieved outstanding performance with AUC 97.0%, sensitivity: 93.28%, specificity: 91.38%, PPV 94.46%, and NPV 90.06 (all scores cross-validated with k = 5) as indicated in Table 5. Figure 11 shows the cross-validated ROC curve of the CNN model on the augmented dataset.

As a final step, we prepared a mini-batch dataset that encapsulated only single trials from each of 59 participants and evaluated the DNN’s (the best performing model) accuracy. This dataset contains only 59 ETSP images, as the name implies. We used the leave-one-out cross-validation (LOOCV) technique to train and validate the model. LOOCV is recommended over k-fold cross-validation; in this method, one randomly selected ETSP image from the minibatch of 59 serves as the test image, while the rest of the photos serve as the training set. The procedure is repeated 59 times, with each run having 50 epochs with a batch size of 16. The resulting scores are in Table 6.

## 4. Discussion

Diagnosis of ASD is still a challenging task for clinicians due to heterogeneity in its symptoms. Current methods of ASD diagnosis are based on analysis of a child’s behavior, language skills, cognitive functioning at different ages, and a source of information gathered from parents or caretakers. As previously stated, these procedures are highly subjective, leading to misdiagnoses or missed diagnoses. Therefore, identifying an objective biomarker to assist clinicians with a quick and reliable screening of ASD would substantially impact current diagnosis practices. As mentioned previously, this study looked into the feasibility of using eye-tracking data to accurately identify ASD traits in children using optimal ML algorithms. Hence, we evaluated the performance of three classical algorithms and a DNN algorithm using the original and augmented ETSP images. The classical ones were ready-to-use models with minimal settings, making them lightweight and suited for low-power computers. DNN models, on the other hand, are handcrafted models that necessitate high-performance computers with a GPU. We wired multiple DNNs by modifying the CNN module’s kernel settings (size and counts) and the number of deep layers and dropout layers in the DNN module to obtain the best results. 

We found that the BDT model showed a better score than the DSVM and DJ models on the original dataset. The BDT model could discriminate ASD from TD with a mean AUC of 67.6%, detecting 64.40% of true positive cases and 57.40% of true negative cases. However, the findings did not look promising; we reasoned that the retrieved features might not support the model to distinguish between the two classes. In comparison, the DNN model achieved a mean AUC of 78.6%, successfully detected 78.57% true positive cases and 75.47% of true negative cases. The CNN-based feature extractors excelled at extracting subtle features from ETSP images, resulting in more precise classification. However, we found that all models were overfitting, which could be due to two factors: model complexity or the limited size of the dataset. We hypothesized that a larger dataset would yield better results from the same models, so we utilized data augmentation to populate the original dataset and reran the experiments. The resulting scores show that the AUC of the classical and DNN models improved significantly, with the BDT showing a 10% improvement (AUC 74.6%), and the DNN’s improvement was 23.4% (AUC 97.00%), higher than the performance (AUC: 90%) reported in the research work on the same dataset with augmentation [24].

The discrepancy between the results using original and augmented datasets may be due to possible data contamination as similar replicates are entered into the training set, as seen in the testing dataset. To mitigate the above issue, we formed a mini dataset that accommodated only single trials from 59 participants, and we continued experiments using the DNN model. The results show that the model detected 26 true negative cases out of the 30, scoring a specificity of 86.67%, 17 true positive cases out of the 29, offering sensitivity of 58.62%, NPV 64%, and PPV 80.95% with an overall accuracy of 72.88%. The result appears to be promising using such a small dataset. For an extensive dataset containing single trials from thousands of individuals, we predict the DNN model to deliver a superior score.

The above findings support the hypothesis that ET could be a potential biomarker for ASD screening. Using the proposed DNN model, we can expedite a reliable outcome if ET data are valid and authentic. Furthermore, unlike contemporary questionnaire-based ASD screening tools such as QCHAT and M-CHAT, which are prone to subjectivity, screening by ET data provides an objective assessment because it does not require any manual input. According to research studies [47,48,49,50], ASD symptoms are likely to be reflected on ET data in children as young as 6 to 24 months of age, well before ASD-related behavioral traits manifest. So, if we can record ET data accurately from toddlers, we can discover early indicators of ASD in them using the proposed DNN model.

Often, clinicians find it difficult and time-consuming to diagnose ASD because of the heterogeneity in the ASD symptoms. Due to heterogeneity, distinct variations in ASD-relevant symptoms exist within a subclass of ASD [51]. Unfortunately, there is no reliable method for quantifying autism heterogeneity in children that helps plan for effective interventions targeted to small groups rather than a broad range. However, studies have found a strong link between ASD heterogeneity and ET data complexity [52]. Therefore, by leveraging the capabilities of complex CNN-based algorithms, ML models could quantify heterogeneities in ASD symptoms, allowing for accurate ASD severity grading using ET data.

### 4.1. Clinical Implications

Rather than being a diagnostic tool, the research model we proposed in this work is meant to be used for ASD screening using ET data. This methodology would improve the efficacy of current ASD screening methods while simplifying the diagnosing procedure. Furthermore, utilizing the suggested DNN model with ET data, a screening test takes less than a second. With the support of a comprehensive and reliable ETSP image dataset, we could identify autism with high accuracy even at the infancy stage with needed changes in the model’s anatomy. Further, ASD screening using ET data is subjectivity-free, making it more accurate and reliable than manual screening methods. Finally, a mobile app with an incorporated DNN-based ASD classifier and an extensive ET dataset of children aged 6 to 24 months may assist parents in independently screening their toddlers for ASD using a mobile device.

### 4.2. Limitations

Obtaining an extensive dataset representing the complete spectrum of ASD symptoms was the most challenging task in our study. A few open-access ET datasets are available; however, they combine multiple ETSP images from a small set of participants rather than a single trial from a large group, making them unsuitable for reliable ASD screening.

Furthermore, the quality and consistency of the training data determine the accuracy of the proposed ASD screening tool. Hence, the clinician must ensure that the child is staring at the eye tracker throughout the trials and that the recording environment is free from any distractions that may impact the child’s attention.

We would like to investigate the usability of electrooculogram (EOG) to predict autism in children using a machine learning algorithm in our future work. 

## 5. Conclusions

Using the eye-tracking scan path image dataset, we tested four different machine learning algorithms to identify an accurate model for detecting ASD. Using the original dataset and an extended dataset created through augmentation, we trained three traditional ML algorithms and one DNN-based algorithm. Experimental results indicate that the conventional algorithms were found ineffective for this dataset. The DNN-based model outperformed the state-of-the-art models published in recent days. The DNN-based model achieved a cross-validated AUC of 78% on the original dataset, 97% when tested on the augmented dataset. The DNN model scored an accuracy of 72.88% for the mini-batch dataset with only 59 individual trials. Experimental results strongly suggest that using ETSP images and the proposed DNN model, a quick, accurate, and reliable diagnosis of ASD can be performed if the outcomes clinically correlate with results of other commonly used ASD screening tools.

## Figures and Tables

**Table 1:** Summary of the ETSP Image Dataset.

Description	Details
Number of participants	59
Number of classes	2
Number of TD	29
Number of ASD—Diagnosed	30
Total number of ETSP images	547
ETSP Images—TD	328
ETSP Images—ASD	219
Size of image	640 × 480 × 3
CARS Scores (Mean)	32.97
Age in years and Mean age	3–13, 7.88
% Gender distribution (M:F)	64:36

**Table 2:** Details of the datasets used in our study.

Dataset	Total Number of Images	Number of TD. Images	Number of ASD. Images	Size of the Image
Dataset I (Original)	547	328	219	640 × 480 × 3
Dataset II (Augmented dataset)	2566	1519	1041	640 × 480 × 3

**Table 3:** Performance of the ML algorithms’ tests on the original dataset.

Dataset	Model	%Sensitivity	%Specificity	%PPV	%NPV	%AUC
Original	DSVM	44.06	55.55	52.0	47.61	51.50
	DJ	55.93	64.80	63.46	57.37	60.40
	BDT	64.40	57.40	62.20	59.60	67.60
	DNN	78.57	75.47	87.12	62.50	78.00

**Table 4:** Configuration of the best performing DNN.

Layer ID	Type/Function	Dimensions (Number of Kernel and Size)	Dimensions of Output Feature
Level 1	2D convolution Activation-ReLU	16, 3 × 3	16, 100 × 100
	Max pooling	16, 2 × 2	16, 50 × 50
Level 2	2D convolution Activation-ReLU	32, 3 × 3	32, 50 × 50
	Max pooling	32, 2 × 2	32, 25 × 25
Level 3	2D convolution Activation-ReLU	64, 3 × 3	64, 25 × 25
	Max pooling	64, 2 × 2	64, 12 × 12
Level 4	2D convolution Activation-ReLU	128, 3 × 3	128, 12 × 12
	Max pooling	128, 2 × 2	128, 6 × 6
	Flatten Layer	-	1 × 4608
	Dense Layer 1 Dense Layer 2	256 128	-
	Output Layer (Sigmoid Activation)	1	-

**Table 5:** Performance of the ML algorithms’ tests on the augmented dataset.

Dataset	Model	%Sensitivity	%Specificity	%PPV	%NPV	%AUC
Original	DSVM	43.90	72.80	51.51	66.60	61.60
	DJ	38.28	84.15	61.05	67.75	66.80
	BDT	49.80	86.50	70.56	72.66	74.60
	DNN	93.28	91.38	94.46	90.06	97.00

**Table 6:** Perfomance of the DNN’s tests on the mini batch dataset.

Dataset	Model	%Sensitivity	%Specificity	%PPV	%NPV	%AUC
Mini batch	DNN	58.62	86.67	80.95	64.00	72.88

## Data Availability

The dataset used in our research studies is available at https://figshare.com/articles/dataset/Visualization_of_Eye-Tracking_Scanpaths_in_Autism_Spectrum_Disorder_Image_Dataset/7073087, accessed on 12 January 2022. We will provide the Python notebook we developed as part of the research study upon request.
